# ENHANCING PERSON-CENTERED DEMENTIA CARE BY NURSING HOME STAFF USING MICROLEARNING: LITTLE MESSAGE WITH A BIG IMPACT

**DOI:** 10.1093/geroni/igz038.008

**Published:** 2019-11-08

**Authors:** Jenny Inker, Christine J Jensen, Sonya Barsness

**Affiliations:** 1 Virginia Commonwealth University, Richmond, Virginia, United States; 2 Riverside Center for Excellence in Aging and Lifelong Health, Williamsburg, Virginia, United States; 3 Sonya Barsness Consulting, LLC, Arlington, Virginia, United States

## Abstract

Effective training is critical to providing quality care in long-term care environments, where many residents have dementia. Training has been linked to positive resident care outcomes and improved job satisfaction of staff. The aim of this study was to develop, pilot, and evaluate a Microlearning training curriculum, using short (5-10 minute) “bursts” of training available through an online platform on demand (i.e. 24/7). The expected outcomes were to improve staff knowledge, attitudes, and skills regarding person-centered dementia care and to increase job satisfaction. Researchers translated the Centers for Medicare and Medicaid Hand-in-Hand training curriculum into 52 weekly Microlearning lessons delivered via an online platform (accessible by computer, IPad or smart phone) followed by a short quiz. Using pre- and post-tests, nine focus groups, and fourteen telephone interviews, the researchers engaged with a convenience sample of staff (N = 244) working at all levels from direct care to leadership in nine nursing homes in Virginia. Pre- and post-tests comprised items from the Dementia Attitudes Scale and the Nursing Home Nurse Aide Job Satisfaction Scale. Results from a between subjects t-test demonstrated significant improvements in attitudes to people with dementia. Focus groups and interviews revealed high satisfaction with the training with a significant majority agreeing it was a helpful way to learn and that they were able to apply what they had learned to caring for residents. This pilot demonstrates a promising new practice for training long-term care staff. Further research using a control group receiving usual training is indicated.

